# Reduced Frizzled‐Related Protein B Expression Is Associated With Wnt/β‐Catenin Signaling Activation and Calcification in Degenerative Menisci

**DOI:** 10.1002/jor.70295

**Published:** 2026-10-07

**Authors:** Yusuke Inoue, Ken Kumagai, Jisun Kim, Kimi Ishikawa, Hyonmin Choe, Hiroyuki Ike, Naomi Kobayashi, Yutaka Inaba

**Affiliations:** ^1^ Department of Orthopaedic Surgery and Musculoskeletal Science, Graduate School of Medicine Yokohama City University Yokohama Kanagawa Japan; ^2^ Department of Orthopaedic Surgery Yokohama City University Medical Center Yokohama Kanagawa Japan

**Keywords:** calcification, FRZB, meniscus, osteoarthritis, Wnt/β‐catenin signaling

## Abstract

Meniscal calcification is increasingly recognized as a pathological feature associated with the progression of osteoarthritis (OA). However, the molecular mechanisms underlying this process remain poorly understood. This study aimed to investigate the involvement of Wnt/β‐catenin signaling and its extracellular antagonist frizzled‐related protein B (FRZB) in pathological calcification of degenerative human menisci. Human meniscal specimens were obtained from patients with knee OA and non‐OA controls and analyzed histologically and immunohistochemically for calcification and expression of hypertrophic/calcification‐associated markers, β‐catenin, and FRZB. An in vitro calcification model using primary human meniscal cells was established, and the role of FRZB was examined by supplementation with recombinant FRZB or shRNA‐mediated FRZB knockdown. Calcification was assessed with Alizarin Red S staining, and expression of Wnt/β‐catenin signaling‐related and hypertrophic/calcification‐associated genes was analyzed using quantitative PCR and western blotting. Degenerative menisci exhibited prominent calcium deposition accompanied by increased expressions of RUNX2, collagen type X, and MMP‐13. Calcified menisci showed enhanced β‐catenin expression and reduced FRZB expression compared with non‐calcified menisci. In an in vitro model, meniscal cell calcification was accompanied by downregulation of FRZB and activation of Wnt/β‐catenin signaling, along with upregulation of RUNX2, collagen type X, and MMP‐13. Recombinant FRZB attenuated Wnt/β‐catenin signaling activity and reduced calcification, whereas FRZB knockdown enhanced Wnt/β‐catenin signaling and increased calcium deposition. Reduced FRZB expression was associated with enhanced Wnt/β‐catenin signaling activity and increased calcification in degenerative menisci. The findings suggest a potential inhibitory role for FRZB in the context of Wnt/β‐catenin signaling associated meniscal calcification.

## Graphical abstract

Reduced FRZB expression in degenerative menisci activates Wnt/β‐catenin signaling and promotes meniscal calcification through RUNX2 upregulation. This process is associated with increased expression of calcification‐related markers and calcium deposition. These findings support a functional role for FRZB as a negative regulator of Wnt/β‐catenin signaling in the context of meniscal calcification.

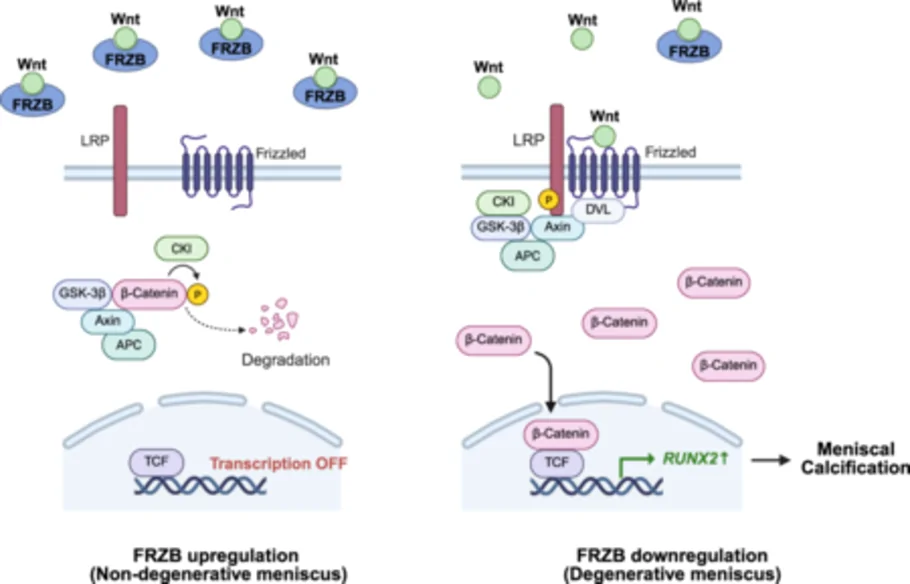

## 1 Introduction

Osteoarthritis (OA) is the most common cause of musculoskeletal impairment and is expected to increase substantially because of population growth and ageing [1, 2]. The meniscus plays a crucial role in protecting articular cartilage by distributing loads across the tibiofemoral joint, absorbing shocks, stabilizing the joint, facilitating lubrication, and supporting nutrition and proprioception [3]. Previous studies have suggested an association between meniscal degeneration and the development of knee OA [4]. Meniscal degeneration has attracted significant attention due to its critical role in the progression of OA.

Meniscal calcification has been reported in degenerative menisci and implicated in the progression of OA, although its prevalence and pathological significance have not yet been fully established [5, 6, 7]. Meniscal calcification in OA is characterized by the predominant deposition of basic calcium phosphate crystals rather than calcium pyrophosphate dihydrate crystals [8, 9]. Although calcification in degenerative joint tissues such as cartilage and ligaments is known to be driven by multiple interacting molecular pathways, among which aberrant activation of Wnt/β‐catenin signaling is a well‐characterized contributor to OA‐associated pathological changes [10, 11, 12], the molecular mechanisms underlying meniscal calcification remain largely unexplored.

Frizzled‐related protein B (FRZB), also known as secreted frizzled‐related protein 3 (SFRP3), is a critical extracellular antagonist of Wnt/β‐catenin signaling that limits aberrant Wnt pathway activation [13, 14]. A recent study reported that FRZB expression is markedly decreased in degenerative human menisci [15]. Based on these observations, it was hypothesized that reduced FRZB expression leads to enhanced Wnt/β‐catenin signaling and thereby contributes to meniscal calcification. However, to date, the role of FRZB and Wnt/β‐catenin signaling in meniscal calcification has not been systematically investigated. The associations of FRZB expression, Wnt/β‐catenin signaling activity, and calcification in degenerative menisci were examined.

## 2 Methods

### 2.1 Sample Collection

Fresh meniscal specimens were obtained from patients with knee OA undergoing total knee arthroplasty (TKA) (OA menisci, *n* = 12, age 74 ± 12 years) and from patients without knee OA undergoing TKA or limb amputation (non‐OA menisci, *n* = 3, age 26 ± 13 years). Patients with a history of knee injury, prior knee surgery, inflammatory arthritis, or joint infection were excluded. This study was approved by the Institutional Review Board of the hospital where the surgeries were performed (Approval No. B191100008), and written, informed consent was obtained from all participants.

### 2.2 Sample Preparation and Histological Evaluation

Samples from the middle segment of each meniscus were cut into 1.5‐cm sections and fixed in 10% neutral buffered formalin, followed by ethanol dehydration. The samples were then embedded in paraffin and radially sectioned at 5 µm thickness. To preserve calcifications, no decalcification was performed. The sections were stained with hematoxylin and eosin (H&E), Safranin‐O‐Fast Green, and von Kossa stain using standard protocols. All samples were evaluated using a microscopic meniscal grading system by Pauli et al [16]. Two independent graders (YI & KI) assessed the samples blindly, and a consensus score was then determined. The grading used in the present study refers to the histological degeneration grade of the meniscus and not to the radiographic or clinical grade of knee OA. For von Kossa quantification, images of stained meniscal sections were analyzed using ImageJ software (National Institutes of Health, Bethesda, MD, USA). The calcification rate was calculated as the von Kossa‐positive calcified area divided by the total meniscal tissue area in each section and expressed as a percentage.

### 2.3 Immunohistochemistry

The serial sections of the specimens used to assess degeneration grade were deparaffinized and rehydrated following standard protocols. Antigen retrieval was performed by heating the sections in citrate buffer (pH 9.0) for 20 min, after which they were incubated with primary antibodies targeting RUNX2 (Abcam, Cambridge, UK), MMP‐13 (Proteintech, Rosemont, IL, USA), β‐catenin (Proteintech), and FRZB (Novusbio, Centennial, CO, USA) at room temperature for 1 h. For antigen retrieval prior to incubation with antibodies against collagen type X (LSBio, Seattle, WA, USA), hyaluronidase digestion was performed for 30 min. Endogenous peroxidase activity was blocked by treating the sections with 3% hydrogen peroxide in methanol for 30 min. The sections were then incubated with immunoperoxidase polymer (Histofine Simple Stain MAX‐PO MULTI; Nichirei, Tokyo, Japan), followed by color development using DAB (ImmPACT DAB peroxidase substrate; Vector Laboratories, Newark, CA, USA). The sections were counterstained with hematoxylin. Photomicrographs were captured using a Keyence BZ800 microscope (Keyence, Osaka, Japan). Immunopositivity was assessed by counting the number of positive cells and calculating the positive‐to‐total cell ratio in six different high‐power fields of each section. For each section, three fields were selected from the inner zone and three fields from the outer zone. Medial and lateral menisci were not analyzed separately.

### 2.4 Primary Cell Isolation and Culture Conditions

Meniscal cells were isolated from patients with knee OA (*n* = 6) by treatment with collagenase (MilliporeSigma, St. Louis, MO, USA). Meniscal tissues were collected without separating inner and outer zones. Cells at passage 1 were plated at an initial density of 8 × 10^4^ cells per well in a 12‐well plate. They were cultured in alpha minimum essential medium supplemented with 10% fetal bovine serum and 1% penicillin‐streptomycin at 37°C in 5% CO_2_ for the first 2 weeks. Subsequently, the CO_2_ concentration was reduced to 3% to facilitate calcification. The medium was replaced three times a week. No β‐glycerophosphate, dexamethasone, ascorbic acid, or other osteogenic supplements were added. After reaching confluence, the cells were maintained without passaging throughout the long‐term culture period. For recombinant FRZB supplementation experiments, primary meniscal cells from four independent OA donors were cultured under the same mineralization‐promoting conditions and treated with recombinant FRZB protein (Abcam) at 0 or 100 ng/mL.

### 2.5 Lentiviral‐Mediated shRNA Gene Silencing

To knock down FRZB expression in primary meniscal cells, lentiviral transduction was performed using MISSION shRNA (Merck KGaA, Darmstadt, Germany) following the manufacturer's instructions. This experiment was performed using cells from four independent OA donors. Briefly, cells were seeded at a density of 5 × 10^4^ cells per well in a 24‐well plate and transduced with either FRZB‐targeting shRNA (MISSION shRNA TRCN0000014460) or a non‐mammalian control shRNA (MISSION pLKO.1‐puro control plasmid DNA, SHC016V). Transduced cells were then selected with 0.75 µg/ml puromycin in the culture medium.

### 2.6 Alizarin Red S Staining

Cells were fixed with 10% neutral buffered formalin and then stained with Alizarin Red S (PG Research, Tokyo, Japan) for 30 min. After washing the wells with pure water, the plates were photographed. The absorbance at 450 nm of Alizarin Red S dye elution was determined in a microplate reader (Infinite F50, TECAN, Männedorf, Switzerland) according to the manufacturer's instructions. Calcium deposition was quantified based on the absorbance of eluted Alizarin Red S dye. Data were normalized to the control group and expressed as relative Alizarin Red S staining.

### 2.7 RNA Extraction and Real‐Time Polymerase Chain Reaction (PCR)

Total RNA was extracted from cultured cells using FastGene RNA Basic Kit (NIPPON gene, Tokyo, Japan). One microgram of total RNA was then reverse transcribed with ReverTraAce qPCR RT Master Mix (Toyobo, Osaka, Japan). Each PCR reaction mixture contained 1× THUNDERBIRD Probe qPCR Mix (Toyobo), 0.3 mM specific primers, and 20 ng of cDNA. Quantitative real‐time PCR was performed on a CFX96 real‐time PCR detection system (BIO‐RAD, Hercules, CA, USA) using TaqMan gene expression assays (Thermo Fisher, Waltham, MA, USA). Gene expression levels were normalized to glyceraldehyde 3‐phosphate dehydrogenase (GAPDH). The TaqMan gene expression assays used in this study were as follows: *RUNX2* (Hs01047973_m1), *MMP13* (Hs00942584_m1), *COL10A1* (Hs00166657_m1), *CTNNB* (β‐catenin) (Hs00355045_m1), *AXIN2* (Hs00610344_m1), *FRZB* (Hs01556515_m1), and *GAPDH* (Hs02786624_g1).

### 2.8 Protein Extraction and Western Blotting

Cultured meniscal cells were washed with PBS (pH 7.4) and lysed using sample buffer for western blotting. Equal amounts of protein from each lysate were separated by sodium dodecyl sulfate‐polyacrylamide gel electrophoresis (SDS‐PAGE) and transferred to a PVDF membrane (BIO‐RAD). The membrane was blocked with Blocking One (NACALAI TESQUE, INC.; Kyoto, Japan) or 5% BSA (NACALAI TESQUE, INC.) in 0.05% Tween 20/Tris‐buffered saline at room temperature for 1 h, followed by overnight incubation at 4°C with antibodies against FRZB (Novusbio), active (non‐phospho) β‐catenin (Abcam) and β‐actin (Abcam). The blots were subsequently incubated with a secondary antibody for 1 h at room temperature. Immunoreactive proteins were detected using ECL Select Western Blotting Detection Reagent (Cytiva, Marlborough, MA, USA) and exposed to a WSE‐6100H LuminoGraph (Atto Co., Tokyo, Japan) for an optimal duration based on the antibody. Band intensities were quantified using CS Analyzer 4 image analysis software (Atto Co.) and normalized to β‐actin levels.

### 2.9 Statistical Analysis

Statistical analyses were performed using GraphPad Prism version 10.6.1 (GraphPad Software, San Diego, CA, USA). Data are presented as mean ± standard deviation. The Mann–Whitney U test was used to compare two independent groups in analyses of human samples. For multiple group comparisons, one‐way analysis of variance (ANOVA) followed by Dunnett's multiple comparisons test was applied. For time‐course *in vitro* experiments, repeated‐measures one‐way ANOVA with Dunnett's post hoc test was used to evaluate temporal changes within the same samples. Paired two‐tailed Student's *t* test was used for comparisons between matched control and treatment conditions derived from the same donor.

## 3 Results

### 3.1 Immunohistochemistry of Hypertrophic/Calcification‐Associated Markers in Human Menisci

To investigate hypertrophic/calcification‐associated features in degenerative human menisci, immunostaining was performed for RUNX2, MMP‐13, and collagen type X (Figure 1A). OA menisci (grade 4) showed clear immunopositivity for RUNX2, MMP‐13, and collagen type X, whereas such positivity was minimal or absent in non‐OA menisci. The percentage of immunopositive cells was quantified in each degeneration grade and compared with non‐OA controls. RUNX2 and collagen type X were significantly increased in grades 2, 3 and 4, and MMP‐13 was significantly increased in grades 3 and 4 compared with non‐OA menisci. (Figure 1B). These findings indicate that meniscal degeneration is associated with increased expression of hypertrophic/calcification‐associated markers.

**Figure 1:**
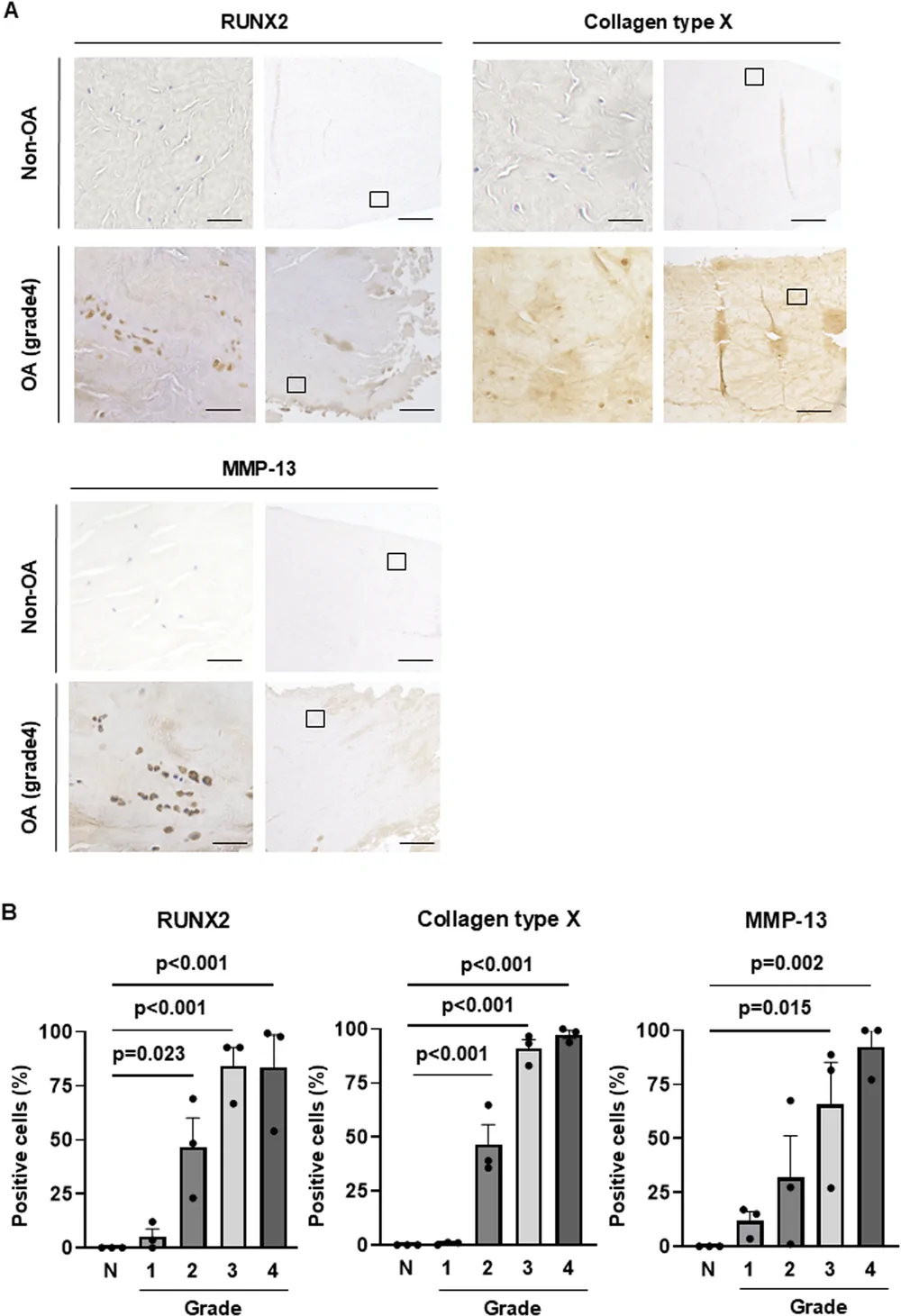
Immunohistochemistry of hypertrophic/calcification‐associated markers (RUNX2, MMP‐13, and collagen type X) in a meniscus. (A) The positive cells (brown) of hypertrophic/calcification‐associated markers are evident in the OA meniscus (grade 4). Boxes in the low‐magnification images indicate the regions shown at higher magnification. (B) Relationships between degeneration grades and cells positive for RUNX2, MMP‐13, and collagen type X. Degeneration was graded using the serial sections. Each dot represents an individual value. OA osteoarthritis. N non‐OA. Bar graphs represent mean ± standard deviation. Scale bars = 50 µm in the high‐magnification images and 500 µm in the low‐magnification images.

### 3.2 Calcification of Degenerative Menisci

To confirm calcification in degenerative menisci, von Kossa staining of human meniscal sections was performed. Calcified regions were defined as areas showing von Kossa‐positive calcium deposition on serial sections, whereas non‐calcified regions were defined as comparable areas without detectable von Kossa‐positive calcium deposition. Calcified areas were rarely observed in non‐OA menisci, whereas calcium depositions were observed in a subset of advanced degenerative menisci (grade 4) (Figure 2A). Quantitative analysis demonstrated that the calcification rate was higher in grade 4 menisci than in non‐OA controls (*p* = 0.01) (Figure 2B). These findings confirm that meniscal calcification is a prominent pathological feature of advanced meniscal degeneration.

**Figure 2:**
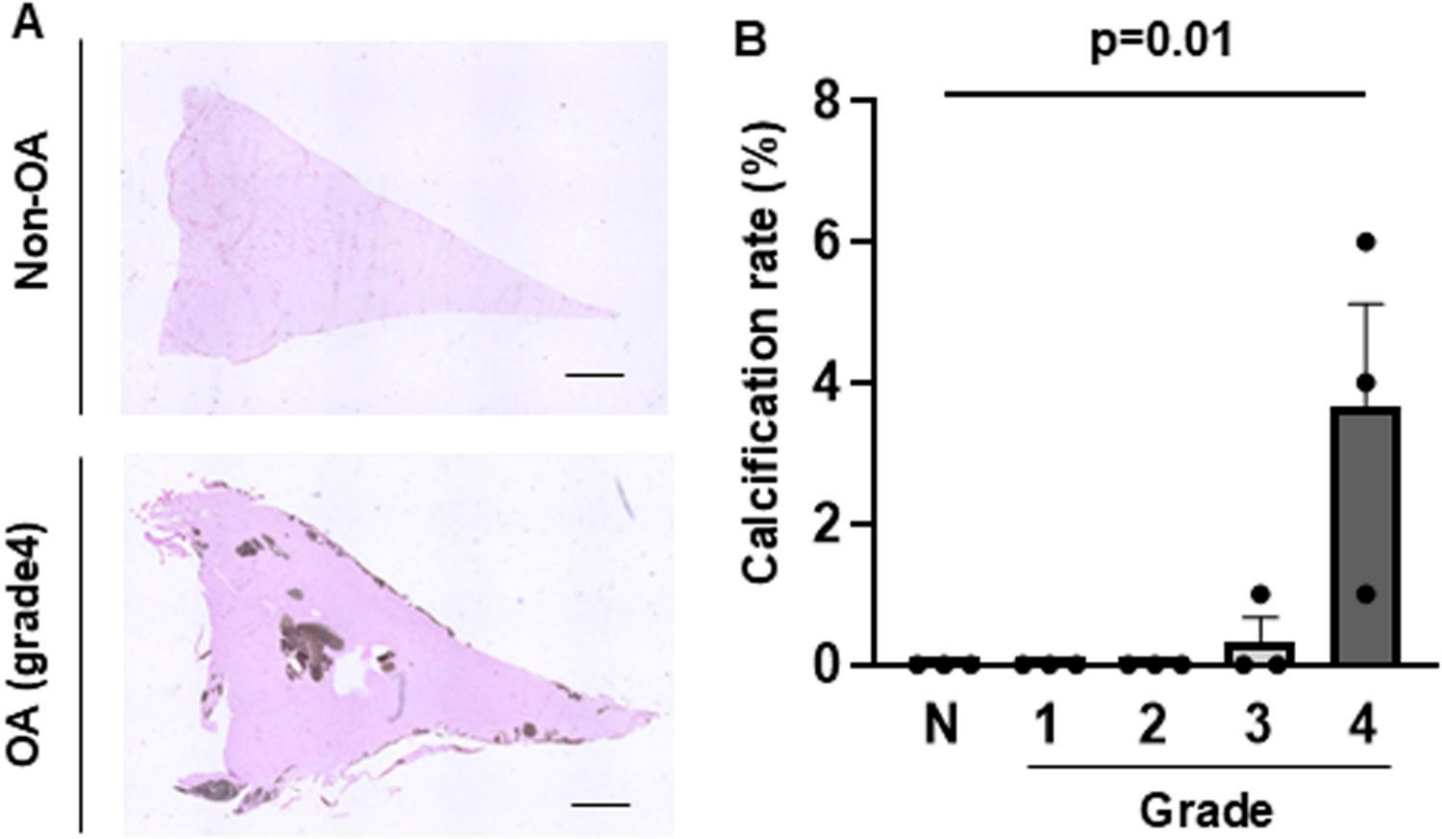
Calcification analysis of a meniscus. (A) Whole sections with von Kossa staining. (B) Percentage of calcification area in menisci from non‐osteoarthritis and osteoarthritis patients. Degeneration of menisci from the osteoarthritis patients was graded using the serial sections. Meniscal degeneration was graded histologically using the Pauli grading system. Each dot represents an individual value. OA, osteoarthritis. N, non‐OA. Bar graphs represent mean ± standard deviation. Scale bars = 2 mm.

### 3.3 Immunohistochemistry of FRZB and β‐catenin in Calcified Menisci

Given the prominent calcification observed in advanced degenerative menisci, the immunohistochemical expression patterns of FRZB and β‐catenin in calcified versus non‐calcified menisci were examined next (Figure 3A). Calcified menisci exhibited greater expression of β‐catenin (*p* = 0.011) and lower expression of FRZB (*p* = 0.007) than non‐calcified menisci (Figure 3B). Therefore, meniscal calcification was associated with increased β‐catenin expression and decreased FRZB expression.

**Figure 3:**
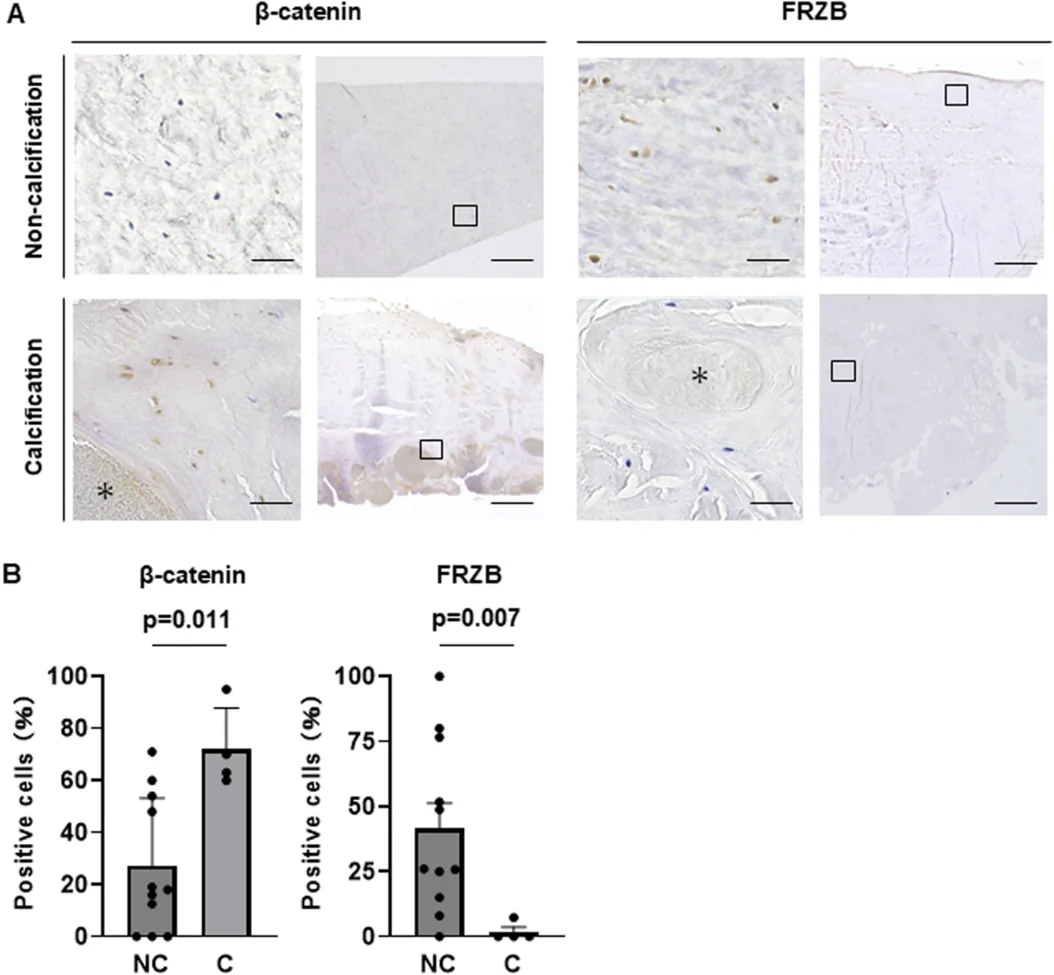
Immunohistochemistry of β‐catenin and FRZB in a meniscus. (A) Cells positive (brown) for β‐catenin are evident in the calcified meniscus, whereas FRZB is apparent in the non‐calcified meniscus. Boxes in the low‐magnification images indicate the regions shown at higher magnification. Asterisks indicate calcified regions identified on corresponding serial sections. (B) Percentage of β‐catenin or FRZB‐positive cells. NC, non‐calcified meniscus. C, calcified meniscus. Each dot represents an individual value. Bar graphs represent mean ± standard deviation. Scale bars = 50 µm in the high‐magnification images and 500 µm in the low‐magnification images.

### 3.4 In Vitro Human Meniscal Cell Model for Studying Calcification

The mechanisms underlying meniscal calcification and the role of FRZB were further investigated using an *in vitro* culture system of human meniscal cells. Compared to 1‐week cultivation, FRZB expression was continuously downregulated at 4 and 6 weeks, whereas expressions of *CTNNB, AXIN2, RUNX2*, and *COL10A1* were continuously upregulated at 4 and 6 weeks, and *MMP13* was upregulated at 6 weeks compared with 1 week (Figure 4A). Alizarin Red S staining was evident at 8 weeks (Figure 4B). These *in vitro* data suggest that upregulation of Wnt/β‐catenin signaling and downregulation of FRZB are associated with calcification of meniscal cells.

**Figure 4:**
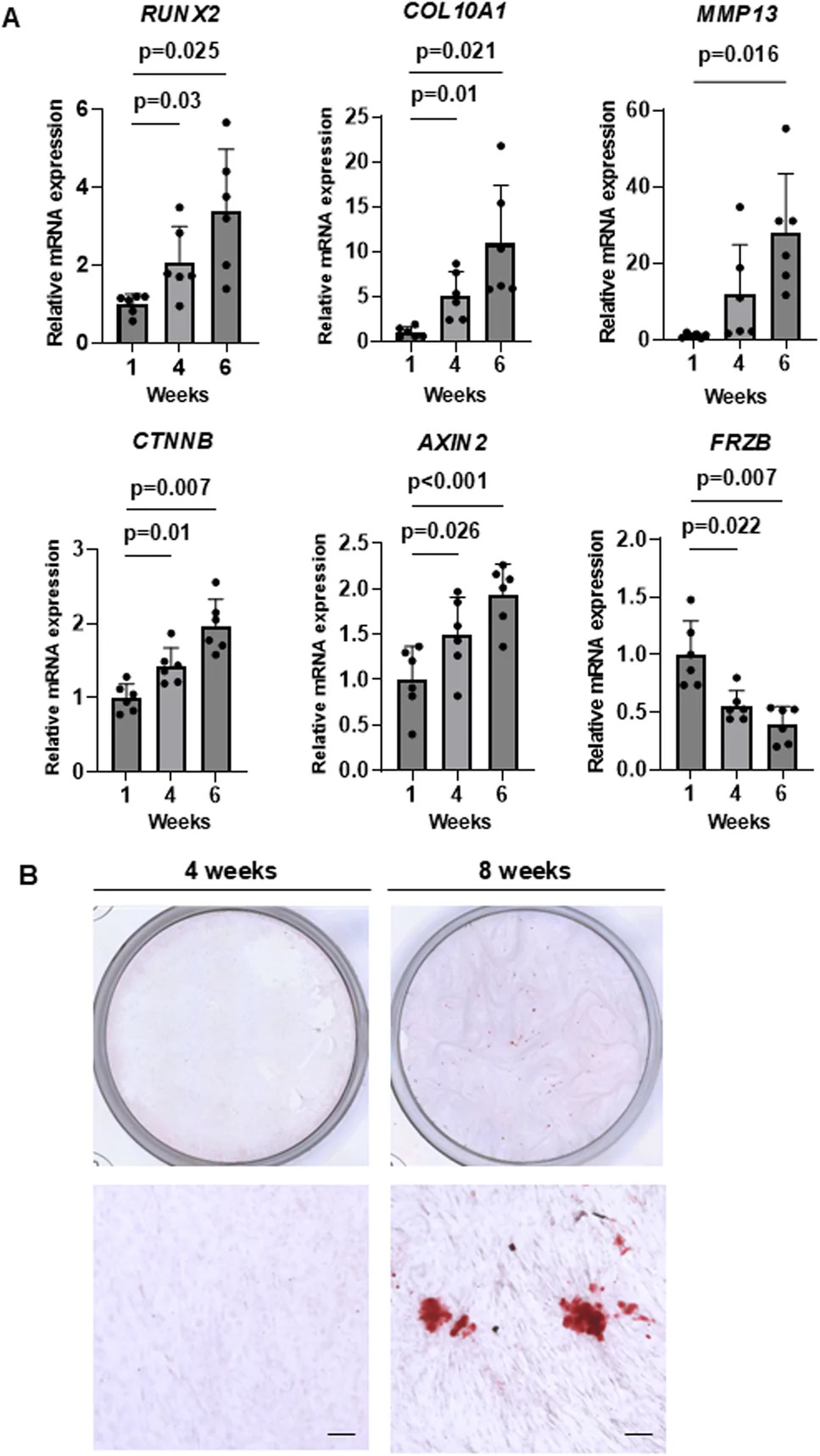
*In vitro* calcification model of human meniscal cells. (A) mRNA expression of hypertrophic/calcification‐associated markers (*RUNX2, COL10A1, MMP13*) and Wnt‐related markers (*CTNNB, AXIN2, FRZB*) at 1, 4, and 6 weeks. Each dot represents an individual value. Bar graphs represent mean ± standard deviation. (B) Photomicrographs of Alizarin Red S staining after 4 and 8 weeks. Whole area of a culture well (upper panels) and magnified area (lower panels). Scale bars = 100 µm. n = 6 independent OA donors.

### 3.5 Effects of FRZB on Meniscal Calcification

To determine whether FRZB directly suppresses meniscal calcification, the effects of exogenous FRZB on calcification in cultured human meniscal cells were examined next. The expressions of *RUNX2* (*p* = 0.043), *COL10A1* (*p* = 0.001), *MMP13* (*p* = 0.033), *CTNNB* (*p* = 0.048), and *AXIN2* (*p* = 0.011) were decreased by addition of FRZB (Figure 5A). Moreover, Alizarin Red S staining was significantly reduced when FRZB was added (*p* = 0.039) (Figure 5B). These results suggest that FRZB attenuates the expression of Wnt/β‐catenin signaling‐related markers, accompanied by decreased expression of calcification‐related markers and reduced calcium deposition.

**Figure 5:**
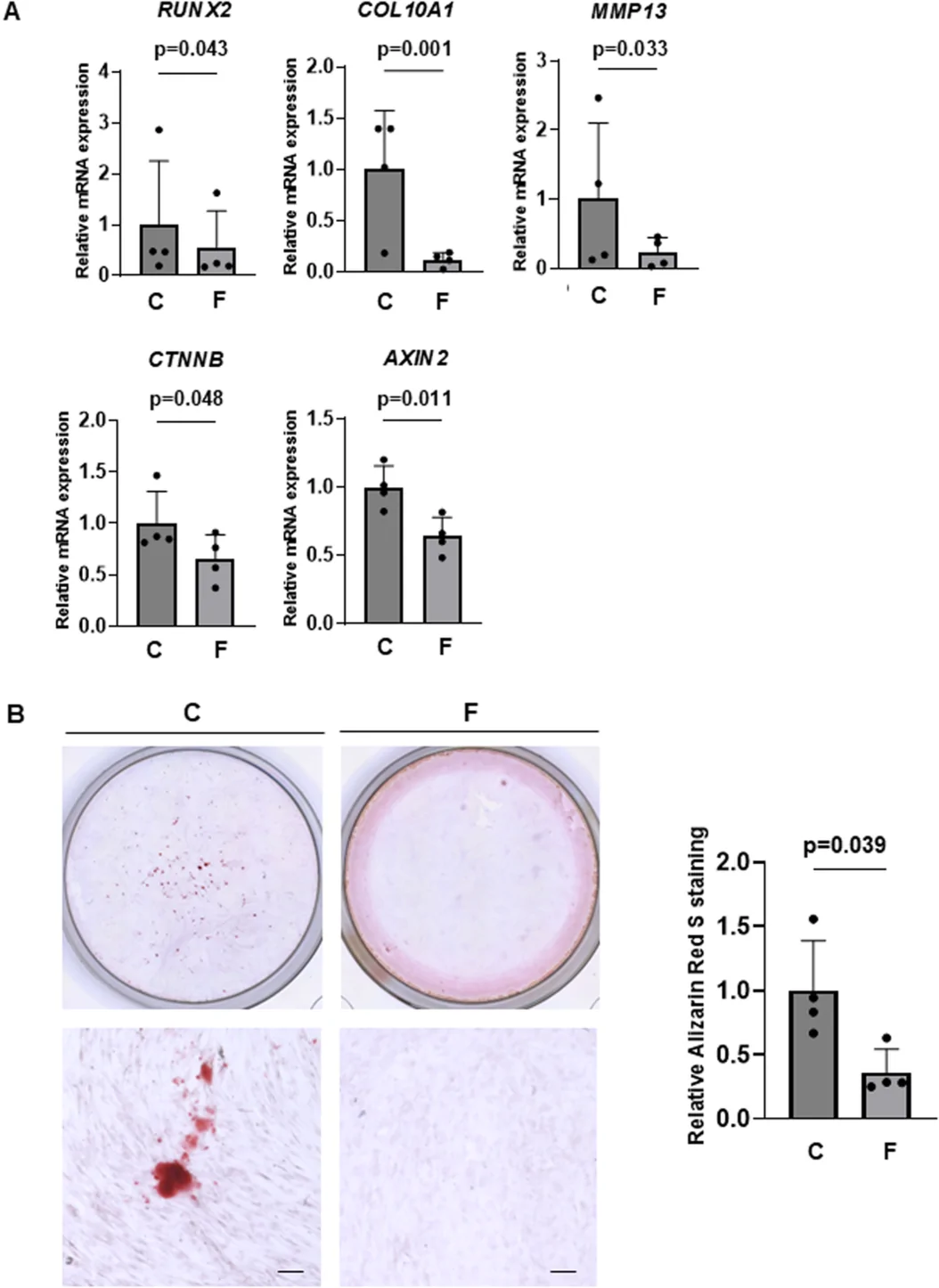
Effect of FRZB on the calcification of human meniscal cells cultured for 8 weeks. (A) mRNA expression of *RUNX2, COL10A1, MMP13, CTNNB*, and *AXIN2*. (B) Photomicrographs of Alizarin Red S staining. Upper panels show the whole area of a culture well, and lower panels show higher magnification images. C, control. F, FRZB (100 ng/ml). Each dot represents an individual value. Bar graphs represent mean ± standard deviation. Scale bars = 100 µm. *n* = 4 independent OA donors.

### 3.6 Effects of FRZB Knockdown on Meniscal Calcification

To further assess the inhibitory effects of FRZB on meniscal calcification, a loss‐of‐function approach was performed using shRNA‐mediated knockdown of FRZB in human meniscal cells. FRZB knockdown achieved a 65% (95%CI 49% to 78%) reduction in FRZB expression and resulted in increased expressions of *RUNX2* (*p* = 0.012), *COL10A1* (*p* = 0.007), *MMP13* (*p* = 0.014), *CTNNB* (*p* = 0.035), and *AXIN2* (*p* = 0.023) (Figure 6A). Western blot analysis confirmed decreased FRZB protein levels (*p* = 0.005) and increased active β‐catenin protein levels (*p* = 0.023) in FRZB‐knockdown cells (Figure 6B). The FRZB‐knockdown group showed significantly greater Alizarin Red S staining intensity (*p* < 0.001) (Figure 6C). These findings support a potential role of FRZB in suppressing meniscal cell calcification, with a concomitant reduction in the expression of Wnt/β‐catenin signaling‐related markers.

**Figure 6:**
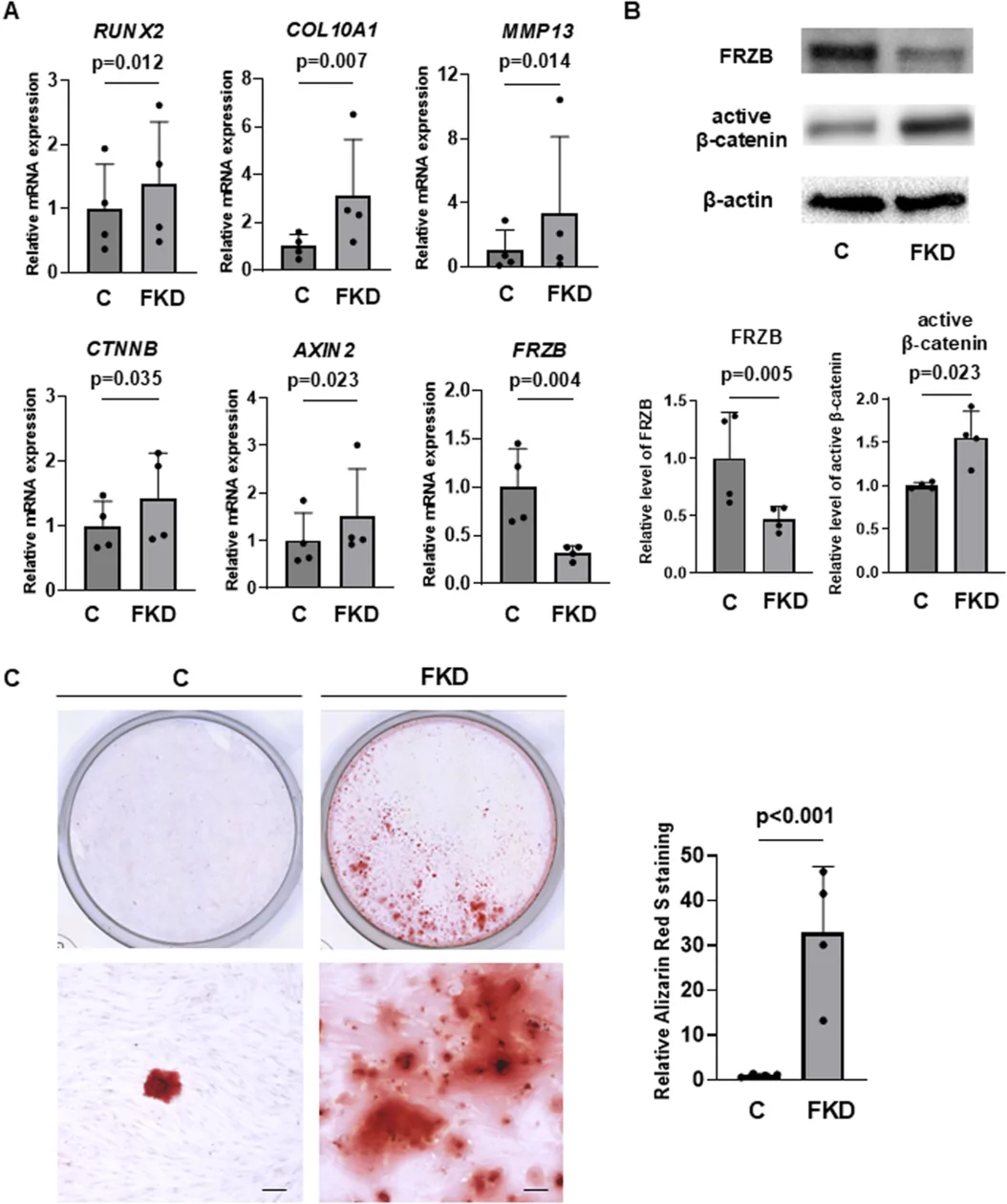
Effect of FRZB gene silencing on the calcification of human meniscal cells cultured for 8 weeks. (A) mRNA expression of *RUNX2, COL10A1, MMP13, CTNNB, AXIN2*. (B) Western blot analysis of FRZB, active β‐catenin, and actin protein expression. (C) Alizarin Red S staining. Upper panels show the whole area of a culture well, and lower panels show higher magnification images. C, control. F‐KD, FRZB knockdown. Each dot represents an individual value. Bar graphs represent mean ± standard deviation. Scale bars = 100 µm. *n* = 4 independent OA donors.

## 4 Discussion

This study demonstrated that Wnt/β‐catenin signaling is closely associated with calcification in degenerative menisci. Human OA menisci exhibited increased β‐catenin activity, with increased expression of hypertrophic/calcification‐associated markers, and calcium deposition was observed in a subset of advanced degenerative menisci. In addition, reduced FRZB expression was associated with enhanced β‐catenin–related marker expression and calcification, whereas recombinant FRZB attenuated these changes. These findings suggest an inhibitory role for FRZB in Wnt/β‐catenin–associated meniscal calcification.

To date, no generally accepted prevalence of meniscal calcification in knee OA has been established. This is likely because previous studies have differed considerably in patient populations, disease severity, anatomical sampling sites, and methods used to detect calcification. A recent histological study detected calcification in 35 of 40 menisci (87.5%) obtained from patients with end‐stage OA [8]. However, posterior horn samples from both the medial and lateral menisci were examined, whereas the present study evaluated the middle segment of the menisci and detected von Kossa‐positive calcification in 4 of 12 OA menisci (33.3%). Because different anatomical regions were analyzed, these findings are not directly comparable. The available evidence suggests that calcification varies among OA menisci and is heterogeneously distributed within the meniscus. Therefore, meniscal calcification should be regarded as one of several pathological changes that may occur during meniscal degeneration, rather than as a universal pathological feature of knee OA.

The present findings extend the current understanding of the molecular features associated with calcification in degenerative menisci. Histological and immunohistochemical analyses showed marked expressions of RUNX2, collagen type X, and MMP‐13, with calcium deposition in a subset of advanced degenerative menisci. RUNX2 is a well‐established transcription factor that promotes the expression of COL10A1 and MMP‐13, which are widely used as markers of hypertrophic and calcification‐associated molecular changes [17, 18, 19, 20]. These markers were selected because degenerative menisci exhibit chondrocyte‐like features [21], and meniscal calcification may share aspects of the hypertrophic differentiation and calcification processes observed in chondrocytes. Abnormal mechanical stress or inflammation in the meniscus has been reported to induce upregulation of collagen type X and MMP‐13 [22, 23]. Consistent with previous reports, the present findings support the involvement of RUNX2 in hypertrophic/calcification‐associated molecular changes observed in degenerative menisci.

Activation of Wnt/β‐catenin signaling is known to induce RUNX2 expression, thereby promoting matrix calcification [24, 25]. However, whether Wnt/β‐catenin signaling is involved in meniscal calcification has not been fully elucidated. Aberrant Wnt/β‐catenin activation has been associated with RUNX2‐dependent pathological calcification in several tissues outside menisci, including the vasculature and tendons. For example, in atherosclerotic arteries, canonical Wnt/β‐catenin signaling induces RUNX2 expression in vascular smooth muscle cells and promotes osteogenic differentiation and vascular calcification [25, 26, 27]. In tendons, activation of canonical Wnt signaling has been reported in tendinopathy. Increased expression of Wnt pathway mediators, including β‐catenin, has been observed in the healing fibroblast‐like cells, chondrocyte‐like cells, and ossified deposits in both animal models and human samples, and Wnt3a stimulation promotes osteogenic differentiation and mineralization of tendon‐derived stem cells [28]. Similarly, the present immunohistochemical analysis demonstrated increased β‐catenin expression concomitant with elevated expression of hypertrophic/calcification‐associated markers in calcified menisci. Collectively, these observations suggest that Wnt/β‐catenin signaling contributes to hypertrophic/calcification‐associated molecular changes in menisci.

FRZB functions as a secreted inhibitor of Wnt/β‐catenin signaling by binding extracellular Wnt ligands and blocking ligand–receptor interactions [13, 14]. Given that FRZB has been shown to suppress Wnt/β‐catenin signaling and inhibit calcification in cartilage [29, 30, 31], this study focused uniquely on FRZB as a suppressor of Wnt/β‐catenin signaling activity and examined its previously unexplored association with meniscal calcification. To further examine the involvement of FRZB in hypertrophic/calcification‐associated responses, we used a simplified calcification culture system with primary human meniscal cells. Calcification of meniscal cells was accompanied by activation of Wnt/β‐catenin signaling, reduced FRZB expression, and increased expressions of RUNX2, MMP‐13, and collagen type X. Notably, these molecular alterations closely mirrored the immunohistochemical findings observed in calcified human meniscal samples. Furthermore, suppression of FRZB enhanced the expression of Wnt/β‐catenin signaling–related and hypertrophic/calcification‐associated markers, whereas supplementation with recombinant FRZB attenuated Wnt/β‐catenin activation, reduced the expression of hypertrophic/calcification‐associated markers, and decreased calcium deposition. Accordingly, these results suggest a potential role for FRZB in limiting Wnt/β‐catenin signaling–associated meniscal calcification.

An *in vitro* chondrocyte mineralization model established by Shukunami et al. [32] was applied in the present study to investigate calcification‐associated responses in primary human meniscal cells. In this culture system, reduction of the CO_2_ concentration from 5% to 3% in the absence of β‐glycerophosphate or other osteogenic supplements promotes matrix mineralization of chondrocytes during endochondral differentiation [32]. Using this experimental model, Wnt/β‐catenin signaling has been shown to promote chondrocyte hypertrophy and matrix mineralization [33, 34]. Degenerative human menisci have previously been reported to acquire chondrogenic characteristics [21]. In the present study, increased expression of hypertrophic chondrocyte‐associated markers, including RUNX2, collagen type X, and MMP‐13, was observed together with calcium deposition and activation of Wnt/β‐catenin signaling in degenerative menisci. These findings suggest that meniscal calcification is accompanied by hypertrophic chondrocyte‐like changes and may share molecular features with the hypertrophic differentiation and matrix mineralization processes that occur during endochondral ossification. Based on these biological similarities, meniscal calcification was investigated using the same *in vitro* model originally developed for endochondral ossification. To date, no established *in vitro* model specifically designed to investigate calcification of primary human meniscal cells has been reported. The present culture system was used as an exploratory proof‐of‐concept model to examine the associations among hypertrophic differentiation, Wnt/β‐catenin signaling, and calcification in meniscal cells. Under these conditions, calcium deposition was accompanied by increased expression of hypertrophic/calcification‐associated markers, activation of Wnt/β‐catenin signaling, and reduced FRZB expression. These molecular changes were consistent with those observed in calcified human meniscal tissues. Although originally developed for chondrocytes, this model provided a biologically relevant framework for testing the hypothesis that meniscal calcification recapitulates aspects of endochondral ossification. However, this experimental system should be regarded as a pilot model rather than a physiological model of meniscal calcification. Because the culture conditions are highly simplified, only part of the pathological process occurring *in vivo* is likely to be reproduced. Long‐term culture under reduced CO_2_ conditions may promote calcium deposition through alterations in the culture environment, cellular stress, or culture‐associated phenotypic changes. Moreover, the complex joint environment, including abnormal mechanical loading, inflammatory mediators, and immune cell interactions, is not fully represented [35, 36, 37]. Further studies using more physiologically relevant experimental models will therefore be required to validate these findings.

Accumulating evidence indicates that intra‐articular calcification is associated with accelerated disease progression in OA [38, 39, 40]. In this context, the present findings suggest that FRZB‐mediated downregulation of Wnt/β‐catenin signaling is linked to calcification in degenerative menisci, which may be relevant to OA progression. However, the clinical implications of this pathway remain uncertain and require further validation, including studies that address causal relationships and disease relevance *in vivo*. Polymorphisms in the *FRZB* (*SFRP3*) gene have been associated with OA [41], and *FRZB*‐deficient mice exhibit increased articular cartilage damage in several experimental models [42, 43]. Taken together, these observations raise the possibility that FRZB may be relevant to the maintenance of joint tissue homeostasis, although its contribution to meniscal pathology and OA progression remains to be established.

Several limitations should be noted. First, the non‐OA control group was small and substantially younger than the OA group. The small sample size limits statistical power in comparisons between groups. Furthermore, because aging itself may contribute to meniscal calcification and alterations in Wnt/β‐catenin signaling, the present human tissue findings should not be interpreted as demonstrating OA‐specific mechanisms. Second, primary meniscal cells were not fully characterized with respect to their phenotype, and culture‐related hypertrophic drift due to dedifferentiation cannot be excluded. However, the use of patient‐derived human meniscal cells provides direct pathological relevance that cannot be achieved in standard rodent systems. Third, a single concentration of recombinant FRZB was used, and dose‐dependent effects were not examined. Therefore, the optimal concentration of FRZB and the dose dependency of its effects on Wnt/β‐catenin signaling and calcification remain unclear, limiting the interpretation of its functional effects. Fourth, the shRNA‐mediated knockdown of FRZB achieved approximately 65% reduction in expression. Although significant effects on hypertrophic/calcification‐associated markers were observed, interpretation of these findings should be made with caution given the incomplete knockdown efficiency. Lastly, the long‐term effects and safety of modulating FRZB *in vivo* remain to be elucidated, and future studies using large‐animal models will be necessary to validate therapeutic applicability.

This study clarified associations of Wnt/β‐catenin signaling, FRZB expression, and meniscal calcification in degenerative menisci and offers insight into the molecular basis of calcification‐related changes in the meniscus. Reduced FRZB expression was associated with enhanced Wnt/β‐catenin signaling activity and increased calcification in both human meniscal tissues and an *in vitro* meniscal cell model. Together, these findings suggest a potential inhibitory role for FRZB in the context of Wnt/β‐catenin signaling‐associated meniscal calcification.

## Author Contributions

YI (Inoue) and Ken Kumagai designed the study. YI (Inoue), Kimi Ishikawa, and Jisun Kim conducted the experiments. YI (Inoue) and Kimi Ishikawa collected the data. YI (Inoue), Ken Kumagai, Kimi Ishikawa, Jisun Kim, Hyonmin Choe, Hiroyuki Ike, Naomi Kobayashi, and YI (Inaba) analyzed and interpreted the data. YI (Inoue) drafted the manuscript, and Ken Kumagai critically revised the manuscript. Ken Kumagai was responsible for the integrity of the data analysis. All authors read and approved the final manuscript.

## Conflicts of Interest

The authors declare no conflicts of interest.

## Data Availability

The data that support the findings of this study are available from the corresponding author upon reasonable request.
